# Deep learning versus parametric and ensemble methods for genomic prediction of complex phenotypes

**DOI:** 10.1186/s12711-020-00531-z

**Published:** 2020-02-24

**Authors:** Rostam Abdollahi-Arpanahi, Daniel Gianola, Francisco Peñagaricano

**Affiliations:** 1 Department of Animal Sciences, University of Florida, Gainesville, FL USA; 2 Departments of Animal Sciences and Dairy Science, University of Wisconsin-Madison, Madison, WI USA; 3 University of Florida Genetics Institute, University of Florida, Gainesville, FL USA

## Abstract

**Background:**

Transforming large amounts of genomic data into valuable knowledge for predicting complex traits has been an important challenge for animal and plant breeders. Prediction of complex traits has not escaped the current excitement on machine-learning, including interest in *deep learning* algorithms such as multilayer perceptrons (MLP) and convolutional neural networks (CNN). The aim of this study was to compare the predictive performance of two deep learning methods (MLP and CNN), two ensemble learning methods [random forests (RF) and gradient boosting (GB)], and two parametric methods [genomic best linear unbiased prediction (GBLUP) and Bayes B] using real and simulated datasets.

**Methods:**

The real dataset consisted of 11,790 Holstein bulls with sire conception rate (SCR) records and genotyped for 58k single nucleotide polymorphisms (SNPs). To support the evaluation of deep learning methods, various simulation studies were conducted using the observed genotype data as template, assuming a heritability of 0.30 with either additive or non-additive gene effects, and two different numbers of quantitative trait nucleotides (100 and 1000).

**Results:**

In the bull dataset, the best predictive correlation was obtained with GB (0.36), followed by Bayes B (0.34), GBLUP (0.33), RF (0.32), CNN (0.29) and MLP (0.26). The same trend was observed when using mean squared error of prediction. The simulation indicated that when gene action was purely additive, parametric methods outperformed other methods. When the gene action was a combination of additive, dominance and of two-locus epistasis, the best predictive ability was obtained with gradient boosting, and the superiority of deep learning over the parametric methods depended on the number of loci controlling the trait and on sample size. In fact, with a large dataset including 80k individuals, the predictive performance of deep learning methods was similar or slightly better than that of parametric methods for traits with non-additive gene action.

**Conclusions:**

For prediction of traits with non-additive gene action, gradient boosting was a robust method. Deep learning approaches were not better for genomic prediction unless non-additive variance was sizable.

## Background

Quantitative genetics theory was established a century ago when Sir Ronald Fisher introduced the infinitesimal model [1]. Theory was mainly developed in the absence of directly observable genotypic data and persisted for decades. However, with the advent of DNA sequencing technologies, the understanding of the genetic background of complex traits has increased. Using the large amounts of molecular genetic data that are currently collected, several studies indicated that epistasis is pervasive in agricultural species [2–4]. However, for prediction of complex traits, the additive model is typically a default assumption in conventional statistical methods since additivity is a close approximation in most cases. Nevertheless, some methods free of assumptions about the genetic architecture of loci that underlie complex traits have been suggested for the prediction of complex phenotypes. These methods include machine-learning techniques and genetic algorithms [5–7]. Machine-learning methods focus on prediction without using a pre-conceived model. On the other hand, conventional statistical approaches formalize relations between variables in the form of explicit mathematical models with parameters that are interpretable in the context of some theory.

Machine learning is increasingly used to deal with problems in analyzing big data and in situations where the number of parameters is much larger than the number of observations. Machine learning has been extensively applied in image processing data, audio recognition and text mining, and the learning algorithms are model specification free and may capture unforeseen information from high-throughput datasets [8]. This is appealing in genomic association studies where important signals may be clustered within genic regions composed of upstream and downstream UTR, introns and exons. The boundaries between genic regions are determined by patterns in the nucleotide sequences. Moreover, interaction between loci is prevalent and recombination hotspots are not uniformly distributed across the genome. Some advanced machine-learning algorithms such as ensemble methods and deep learning (DL) algorithms might help in genome-enabled prediction.

Ensemble methods, such as random forests (RF) [9] and boosting [10], are appealing machine-learning alternatives to conventional statistical methods to analyze complex traits using high-density genetic markers. Indeed, these methods have been already used in genomic prediction using both real and simulated datasets [8, 11, 12]. Boosting and RF are model specification free and may account for non-additive effects. Moreover, they are fast algorithms, even when handling a large number of covariates and interactions and can be used in both classification and regression problems.

Deep learning (DL) is a subset of machine-learning procedures that were originally inspired by the structure and function of the brain and essentially describe a class of neural networks with a large number of nodes and layers. In genomics, DL algorithms have been applied in many areas, largely driven by massive increases in computing power and access to big data. DL algorithms such as the multilayer perceptron (MLP) and convolutional neural network (CNN) might be able to exploit unknown patterns of linkage disequilibrium and of interactions between markers. Recently, some studies have examined DL performance in prediction of complex traits in human and agricultural species [13–16]. Bellot et al. [13] concluded that CNN was competitive to linear models for the prediction of human complex traits, but they did not find any trait where DL outperformed the linear models significantly. Ma et al. [14] reported that DL performed better than genomic best linear unbiased prediction (GBLUP) in prediction of wheat traits. Similarly, Montesinos-López et al. [15] concluded that DL was better than GBLUP when genotype × environment (G × E) interaction was ignored for the prediction of wheat and maize traits. Waldmann [16] using simulation and real pig data found that a shallow MLP performed better than GBLUP and Bayesian LASSO. In short, so far, the evidence does not point to a uniformly better performance of DL methods. Actually, the performance of DL was dismal in some instances examined in Bellot et al. [13].

Most agricultural and human traits have a multifactorial inheritance, with multiple and complex relationships among genes, and between genes with environments. Moreover, linkage disequilibrium across the genome creates ambiguous patterns that complicate the prediction of unobserved phenotypes. Perhaps, DL might be able to better exploit the unknown pattern of disequilibrium among SNPs and capture interaction effects across the genome using large available genotypic and phenotypic data. As such, our objective was to evaluate the predictive ability of two DL methods (MLP and CNN) versus two popular ensemble methods, namely gradient boosting (GB) and RF, with two parametric methods, GBLUP and Bayes B, used as benchmark. The context was whole-genome prediction of real bull fertility with simulations used to supplement the study.

## Methods

### Real dataset

A real dataset consisting of 11,790 US Holstein bulls with sire conception rate (SCR) records was used. The SCR evaluation represents the US national phenotypic evaluation of dairy bull fertility. This evaluation of bull fertility is based on cow field data, i.e., confirmed pregnancy records, and it is considered a phenotypic rather than a genetic evaluation because the fertility estimates include both genetic and non-genetic effects. The current model for evaluating bull fertility considers not only factors related to the bull under evaluation, but also factors (nuisance variables) associated with the cow that receives the unit of semen [17]. The SCR records were obtained from 30 consecutive evaluations provided to the US dairy industry between August 2008 and August 2018. These 30 SCR evaluations are available at the CDCB website (https://www.uscdcb.com/). The estimated genomic heritability of SCR is 0.30 [18]. The reliabilities of the SCR records, calculated as a function of the number of breedings, were also available. For bulls with multiple fertility evaluations, the most reliable SCR record, i.e., the SCR record with the most breedings, was used in the analyses.

Genome-wide SNP data for the US Holstein bulls were kindly provided by the Cooperative Dairy DNA Repository (CDDR). A total of 60,671 SNPs used for genomic evaluation in the US dairy cattle [19] were selected for genetic analysis. SNPs that mapped to chromosome X, had a minor allele frequency lower than 5%, missing rate higher than 5%, and a *P*-value for Hardy–Weinberg disequilibrium less than 10^−6^ were removed from the genotype data using PLINK 2.00 [20]. After quality control, 57,749 SNPs were retained for genomic prediction.

### Simulation dataset

We used stochastic simulation to attain a better understanding of the performance of the deep learning methods under various genetic architectures. A quantitative trait was simulated based on the observed genotypes consisting of 57,749 SNPs from two datasets. The first dataset was composed of the 11,790 individuals with SCR records and the second dataset involved 80,000 genotyped bulls provided by CDDR. To measure the predictive ability of the different methods used, two scenarios of number of quantitative trait nucleotides (QTN) were considered, either small (*n* = 100) or large (*n* = 1000). QTN locations were distributed across the genome in two different ways: (i) clustered QTN randomly sampling one-third of QTN from the SNPs across the genome as core QTN, with two SNPs surrounding each core QTN also treated as QTN, and (ii) randomly located QTN across the genome.

Two scenarios of gene action were simulated: purely additive and a combination of additive, dominance and two-locus epistasis effects. Hereafter, we call the latter as “non-additive gene action”. The additive and non-additive effects were generated as follows.

#### Purely additive action

The allele substitution effects ($\alpha$) were drawn from a standard normal distribution and each was formulated as $\alpha = a + d\left( {q - p} \right)$, where $a$ and $d$ are additive and dominance effects, respectively, and $p$ is the allelic frequency with $q = 1 - p$. In order to produce a purely additive trait, the dominance effect was set to zero. The additive genetic values were calculated by multiplying the genotype codes by the QTN substitution effects and summing over the QTN. The phenotypic value of each individual $i$ ($y_{i}$) was created by adding a normally distributed residual $e_{i} \sim N\left( {0,\sigma_{e}^{2} } \right)$ to the sum over QTN (genetic values) as shown below:

$$\varvec{y}_{\varvec{i}} = \mathop \sum \limits_{{\varvec{k} = 1}}^{\varvec{m}} \varvec{X}_{{\varvec{ik}}}\varvec{\alpha}_{\varvec{k}} + \varvec{e}_{\varvec{i}} ,$$

where *X*_*ik*_ (*i* = 1,.., *n*; *k* = 1,…*m*) is an element of the incidence marker matrix for additive genetic effects ($\alpha_{k}$) and $e_{i}$ is a random residual, where $\sigma_{e}^{2}$ is the residual variance. Genotypes were coded as 0 for “*aa*”, 1 for “*Aa*”, and 2 for “*AA*” to capture additive effects.

#### Non-additive gene action

The simplest type of epistasis is a two-locus model in which each locus has two alleles interacting with each other. Epistasis was simulated only between pairs of QTL including additive × additive (A × A), additive × dominance (A × D), dominance × additive (D × A), and dominance × dominance (D × D) effects. Each QTN interacted with three surrounding QTN. The elements of the incidence matrix (**D**) for modeling dominance effects were equal to 0, 1 and 0 for genotypes “*aa*”, “*Aa*” and “*AA*”, respectively. We simulated overdominance only because incomplete dominance may be partly captured by an additive model, which would not be the case for overdominance.

Once the two loci involved in the interaction were defined, an interaction matrix was created via a Hadamard product of corresponding elements of the additive (**X**) or dominance (**D**) genotype matrices. For instance, a coefficient of 0 was assigned if two genotypes were 0 0 or 0 -, a coefficient of 1 if the two genotypes were 1 1, a coefficient of 2 if the two genotypes were 1 2 or 2 1 and a coefficient of 4 if the two genotypes were 2 2. It should be noted that the final coding for A × D or D × A interaction matrices was 0, 1 and 2, since the genotype code for the dominance matrix was 0 and 1. The codes for the D × D interaction matrix were 0 or 1.

Each pair of interacting loci was assigned four types of interaction effects: (i) $\left( {{\text{A}} \times {\text{A}}} \right){\kern 1pt} {\kern 1pt} aal_{k} l_{{k^{\prime}}}$, (ii) $\left( {{\text{A}} \times {\text{D}}} \right){\kern 1pt} {\kern 1pt} adl_{k} l_{{k^{\prime}}}$, (iii) $\left( {{\text{D}} \times {\text{A}}} \right){\kern 1pt} {\kern 1pt} dal_{k} l_{{k^{\prime}}}$ and (iv) $\left( {{\text{D}} \times {\text{D}}} \right){\kern 1pt} {\kern 1pt} ddl_{k} l_{{k^{\prime}}}$. Here, $l_{k}$ and $l_{{k^{\prime}}}$ represent the $k$ and $k'$ QTN. Each type of epistatic effects was sampled from a gamma distribution with the parameters shown in Table 1. The effect sign was sampled to be positive or negative, each with probability 0.5. The phenotype was created by adding $e_{i}$ to the sum of simulated additive, dominance and epistatic QTN effects:

$$y_{i} = \mathop \sum \limits_{k = 1}^{nQTN} X_{ik} \alpha_{k} + \mathop \sum \limits_{k = 1}^{nQTN} D_{ik} d_{k} + \mathop \sum \limits_{k = 1}^{nQTN - 1} \mathop \sum \limits_{{k^{\prime} = 2}}^{nQTN} aal_{k} l_{{k^{\prime}}} + \mathop \sum \limits_{k = 1}^{nQTN - 1} \mathop \sum \limits_{{k^{\prime} = 2}}^{nQTN} adl_{k} l_{{k^{\prime}}} + \mathop \sum \limits_{k = 1}^{nQTN - 1} \mathop \sum \limits_{{k^{\prime} = 2}}^{nQTN} dal_{k} l_{{k^{\prime}}} + \mathop \sum \limits_{k = 1}^{nQTN - 1} \mathop \sum \limits_{{k^{\prime} = 2}}^{nQTN} ddl_{k} l_{{k^{\prime}}} + e_{i}$$

where $aal_{k} l_{{k^{\prime}}}$, $adl_{k} l_{{k^{\prime}}}$, $dal_{k} l_{{k^{\prime}}}$ and $ddl_{k} l_{{k^{\prime}}}$ are the A × A, A × D, D × A and D × D epistatic effects between QTN $k$ and $k^{\prime}$, respectively. Parameters used for the simulation of additive and non-additive situations are in Table 2. It should be noted that when the number of QTN increases from 100 to 1000, the absolute value of additive effects at each QTN decreases. Thus, additive effects depend on the number of QTN; however, the absolute value of epistatic effects did not depend on the number of QTN. Hence, by increasing the number of QTN, the total epistatic and phenotypic variance increased, but the additive variance was constant. Hence, the narrow sense heritability decreased but broad sense heritability increased.

**Table 1:** Distribution of simulated QTN effects and corresponding parameters

Genetic effects	Number of QTN/Interaction		Distribution
	100	1000	
Additive	100	1000	*N* (*μ* = 0, *σ* = 1)
Dominance	100	1000	*N* (*μ* = 0, *σ* = 0.5)
Additive × Additive	294	2994	Γ (*α *=0.1, *β* = 10)
Additive × Dominance	294	2994	Γ (*α *=0.1, *β* = 10)
Dominance × Additive	294	2994	Γ (* α*=0.1, *β* = 10)
Dominance × Dominance	294	2994	Γ (*α *=0.1, *β* = 10)

*N*: normal; *μ*: mean; *σ*: standard deviation; Γ: gamma; *α*: shape parameter, *β*: scale parameters

**Table 2:** Heritability of traits simulated under additive or non-additive gene action

Gene action	Number of QTN	$h_{a}^{2}$	$h_{d}^{2}$	$h_{I}^{2}$	$H_{{\mathbf{B}}}^{2}$
Purely additive	100	0.30	0.00	0.00	0.30
	1000	0.30	0.00	0.00	0.30
Non-additive	100	0.10	0.10	0.50	0.70
	1000	0.02	0.02	0.68	0.70

Non-additive: mixture of additive, dominance and epistatic effects

$h_{a}^{2}$: additive heritability; $h_{d}^{2}$: dominance heritability; $h_{I}^{2}$: proportion of epistatic variation related to phenotypic variation; $H_{{{B}}}^{2}$: broad sense heritability

### Statistical methods

Four machine-learning algorithms, including two ensemble methods (RF, GB) and two deep learning algorithms (MLP and CNN) were evaluated. The machine-learning algorithms were compared against two standard statistical methods known as GBLUP [21] and Bayes B [22].

#### Conventional statistical methods

*GBLUP*: BLUP is one of the most extensively used regression methods for genomic prediction [21, 22]. The statistical model of GBLUP can be written as:

$${\mathbf{y}} = {\mathbf{1}}\mu + {\mathbf{g}}_{{\mathbf{A}}} + {\mathbf{e}},$$

where ${\mathbf{y}}$ is an n-vector of phenotypes, **1** is an n-vector of ones, $\mu$ is the population mean, ${\mathbf{g}}_{{\mathbf{A}}}$ is a vector of random additive genomic values [${\mathbf{g}}_{{\mathbf{A}}} \sim N\left( {0,{\mathbf{G}}\sigma_{g}^{2} } \right)$] where ${\mathbf{G}}$ ($n \times n$) is the additive genomic relationship matrix between genotyped individuals constructed as $\frac{{{\mathbf{ZZ^{\prime}}}}}{m}$ where ${\mathbf{Z}}$ is the matrix of centered and standardized genotypes for all individuals and $m$ is the number of markers, and $\sigma_{g}^{2}$ is the additive genomic variance, ${\mathbf{e}}$ is the vector of random residual effects [${\mathbf{e}}\sim N\left( {0,{\mathbf{I}}\sigma_{e}^{2} } \right)$] with $\sigma_{e}^{2}$ being the residual variance, and ${\mathbf{I}}$ is the identity matrix. GBLUP was implemented using the BGLR package [23] in the R language/environment, version 3.6.1 [24] as a member of reproducing kernel Hilbert space regression methods [25]. The Gibbs sampler was run for 100,000 iterations, with a 10,000 burn-in period and a thinning interval of 10 iterations, i.e., 9000 samples were used for inference. *Bayes B*: Bayes B is a widely used genomic regression procedure [22], and here we used it together with GBLUP as benchmark against the machine-learning techniques considered. The phenotype of the *i*th individual is expressed as a linear regression on markers:

$$y_{i} = \mu + \mathop \sum \limits_{j = 1}^{m} x_{ij} b_{j} + e_{i} ,$$

where $i = 1 \ldots n$ (individual), $j = 1 \ldots m$ (SNPs), $y_{i}$ is the phenotypic value for individual $i$, $\mu$ is the mean of phenotypes, $x_{ij}$ is an element of the incidence matrix (${\mathbf{X}}$) for marker $j$ and individual $i$, $b_{j}$ is a random effect of marker $j$, and $e_{i}$ is a random residual. In matrix form, the model can be written as: ${\mathbf{y}} = \mu + {\mathbf{Xb}} + {\mathbf{e}}$. Contrary to Bayesian BLUP and Bayes A [22], Bayes B assumes a priori that all markers do not contribute to genetic variation equally. As noted by Gianola [26], Bayes B poses that all markers have a two-component mixture prior distribution. In fact, a given marker has either a null effect with known prior probability, $\pi$, or a $t$ prior distribution with probability $\left( {1 - \pi } \right)$, with $\nu$ degrees of freedom and scale parameter $s^{2}$. The inferences about model unknown parameters were obtained via Gibbs sampling from the posterior distribution. Bayes B was implemented using the BGLR package [23] in the R language/environment, version 3.6.1 [24]. The Gibbs sampler was run for 100,000 iterations, a 10,000 burn-in period and a thinning interval of 10 iterations.

#### Ensemble learning algorithms

*Random forests*: RF is a modification of bootstrap aggregating that builds a large collection of identically distributed trees, and then averages out the results. It takes $B$ bootstrap samples from training data [9] and randomly selects subsets of features as candidate predictors for splitting tree nodes. Each tree minimizes the average loss function in the bootstrapped data and is constructed using the following algorithm:

For $b = 1, \ldots , B$ bootstrap samples $\left\{ {{\mathbf{y}}_{b} ,{\mathbf{X}}_{b} } \right\}$:

1. Draw bootstrap samples of size $N_{train}$ from the training dataset.
2. Grow a random-forest tree $T_{b}$ with the bootstrapped data, by recursively repeating the following steps for each terminal node of the tree, until the minimum node size is reached.
  i. Draw randomly $mtry$ out of the $m$ SNPs.
  ii. Pick the best SNP among the $mtry$ SNPs.
  iii. Split the node into two child nodes.
3. Output the ensemble of trees $\left\{ {T_{b} } \right\}_{1}^{B}$.

The predicted value of testing set ($\hat{y}_{i}$) individual with genotype $\varvec{x}_{i}$ is calculated as $\hat{y}_{i} = \frac{1}{B}\mathop \sum \nolimits_{b = 1}^{B} T_{b} \left( {\varvec{x}_{i} } \right)$. For details on the theory of RF, the readers are referred to Breiman [9] and Waldmann [27].

Three hyperparameters, including number of trees (*ntree*), number of features sampled in each iteration (*mtry*), and number of samples in the final nodes (*nodesize*) must be defined by the user. We assessed various combinations of values of *ntree* = (200, 500, 1000), *mtry* = (500, 1000, 2000, 5000), with the default *nodesize* = 5. The configuration with the minimum out of-bag (OOB) error was *ntree* = 500, *mtry* = 2000 and *nodesize* = 5. The random forest package [28] in the R language/environment, version 3.6.1 [24] was used for implementing RF.

*Boosting*: Boosting is a machine-learning ensemble method that converts weak learners into strong learners, either for classification or regression problems in order to reduce both bias and variance [29]. We implemented XGBoost, which is a popular and efficient form of the gradient boosted trees algorithm. Here, each tree learns from its predecessors and updates the residual errors using the entire dataset. Boosting can also account for interactions between features, automatically select features, and is robust with respect to outliers, missing data and presence of irrelevant features.

Gradient boosting adds new predictors to an ensemble machine sequentially. However, instead of changing the weights for every incorrectly predicted phenotype at each iteration, like AdaBoost [30], the gradient boosted tree method attempts to fit the new predictor to the residual errors made by the previous model. More details on the gradient boosting are in [12, 29–32].

Three hyperparameters must be tuned in boosting: (i) depth of tree, (ii) rate at which the gradient boosting learns, and (iii) number of trees or iterations. The depth of tree and learning rate were determined by five-fold cross-validation. The number of iterations (trees) was determined by examining if the mean squared error in the tuning set had not decreased further during 50 subsequent iterations. We bagged 80% of the training data at each boosting iteration, and the remaining 20% were used as out-of-bag samples. The final value for learning rate was 0.10 and tree depth was 3. We implemented the gradient boosted tree algorithm using the XGBoost package [32].

#### Deep learning algorithms

Deep learning has revolutionized fields such as computer vision, machine translation, and automatic driving, and evaluating its potential for applications in genomics, medicine, and healthcare is an important area of research. There are three common families of supervised DL algorithms: (i) multi-layer perceptron (MLP), (ii) convolutional neural network (CNN) and (iii) recurrent neural network. For a description on each type of network, its assumptions and input features see Goodfellow et al. [33] and Pérez-Enciso and Zingaretti [34]. In this study, we implemented MLP and CNN learning algorithms and a brief explanation of each method is provided below.

### Multi-layer perceptron

**Fig. 1:**
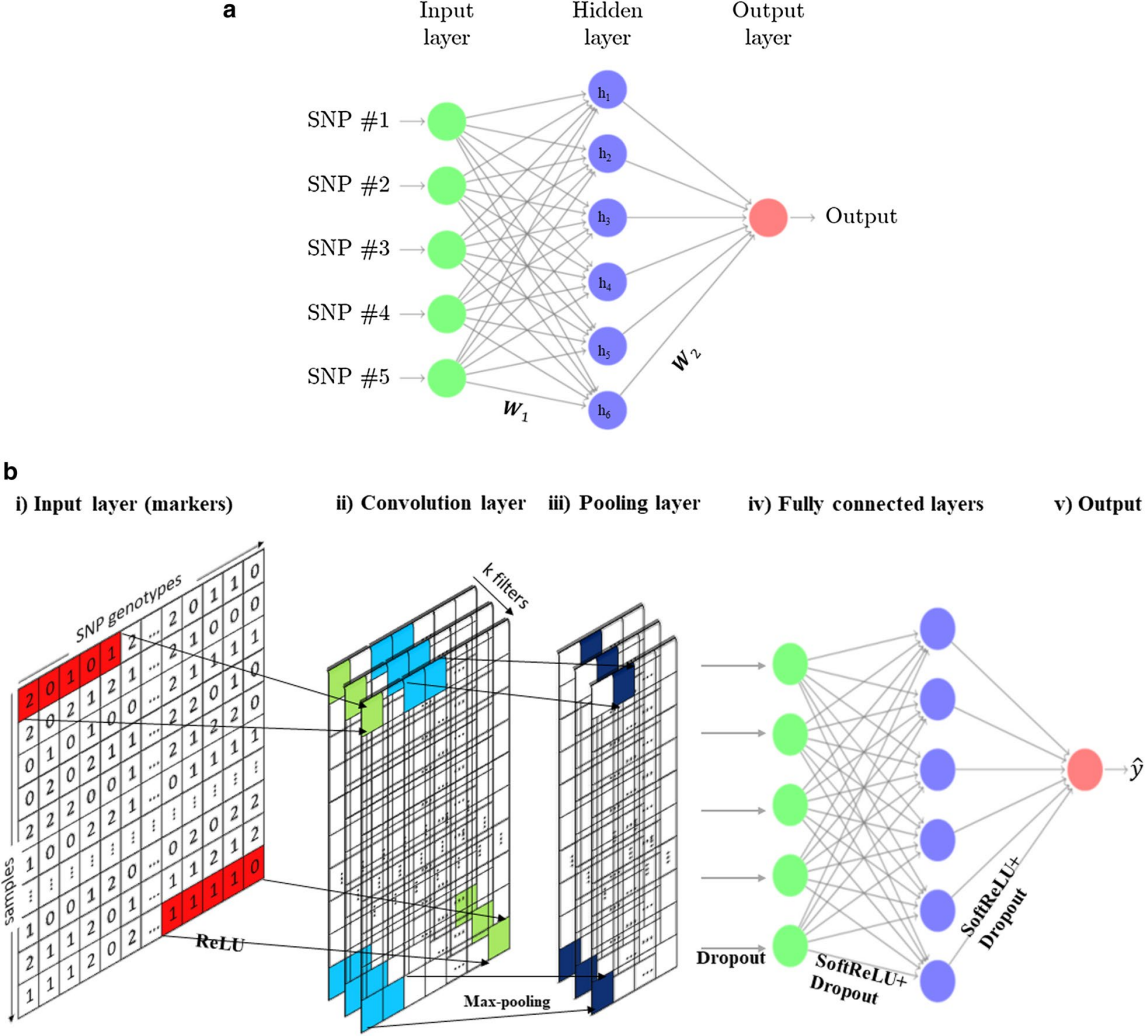
**a** Representation of a multilayer perceptron (MLP) network. Each unit is connected to the units of previous layers by a weighted linear summation, here represented by weight matrices** W**_i_, and an activation function. Redrawn from: http://www.texample.net/tikz/examples/neural-network/. **b** Representation of a convolutional neural network (CNN). (i) The input layer consists of the SNP markers. (ii) Convolution layer consists of *k* filters, which capture the information in input layer by moving filters horizontally with a stride of “s” SNPs. (iii) Pooling layer involves of filters, combining the output of the previous convolution layer at certain locations into a single neuron. (iv) Fully connected layers connect every neuron in previous layer to every neuron in next layer. ‘ReLU’ indicates the rectified linear unit; softReLU indicates smooth rectified linear unit; Dropout indicates the dropout conduct layer

MLP is also known as feed-forward neural network or densely connected neural network. In MLP, the information flows from the input layer to the output layer. The MLP is composed of three types of layers: input layer, hidden layers, and output layer. Figure 1a presents a diagram of a three-layer MLP with five input layer units, six hidden layer units, and one output layer unit. Here, h_1_, h_2_,…, h_6_ are called hidden layer units because they are not directly observed. A single hidden layer MLP model can be represented in the following form:

$${\hat{\text{y}}} =\varvec{\sigma}\left( {{\mathbf{XW}}_{\text{1}} \text{ + }{\mathbf{b}}} \right){\mathbf{W}}_{2} ,$$

where $\hat{\varvec{y}}$ is the vector of predicted observations, ${\mathbf{W}}_{1}$ and ${\mathbf{W}}_{2}$ denote the weight matrices that relate the input genotype matrix ${\mathbf{X}}$ of dimension $n \times p$ to the output layer of ${\mathbf{y}}$ of dimension $n \times 1$ through the hidden layer. The dimension of the ${\mathbf{W}}$ matrices is number of units in the $\left( {k - 1} \right){th}$ layer times number of units in the $k{th}$ layer, where units are neurons and $k$ is the layer number. Parameter $\sigma$ is the activation function modeling the connection between the two consecutive layers and ${\mathbf{b}}$ is the bias (intercept) matrix associated with ${\mathbf{W}}_{1}$ and ${\mathbf{W}}_{2}$. In regression problems, the activation function for connecting the last hidden layer to the output layer is typically chosen to be linear or the Gaussian radial basis function.

For regression problems, the loss function is usually:

$${\mathcal{L}}\left( {y,\hat{y}} \right) = \frac{1}{2n}\mathop \sum \limits_{i = 1}^{n} \parallel y_{i} - \hat{y}\parallel_{2}^{2} ,$$

where $\parallel \cdot \parallel_{2}^{2}$ is the Euclidean squared norm. When the number of predictors (*m*) is larger than the number of observations (*n*), the MLP over-fits the data, and hence, it is required to regularize the MLP parameters **θ** = {**W**_1_, **W**_2_, **b**}. The regularization factors are introduced during optimization. One typical regularization term is the ℓ_2_ penalty through weight decay parameters *λ*_i_, which need to be learned via some search algorithms or cross-validation. Therefore, the loss function to minimize is:

$${\text{minimize}}\left\{ {J\left( {\varvec{\uptheta}} \right) = \frac{1}{2n}\mathop {{\sum }\parallel }\limits_{i = 1}^{n} y_{i} - \hat{y}_{i} \parallel_{2}^{2} + \lambda_{1} \parallel {\mathbf{W}}_{1} \parallel_{2}^{2} + \lambda_{2} \parallel {\mathbf{W}}_{2} \parallel_{2}^{2} + \lambda_{3} \parallel {\mathbf{b}}\parallel_{2}^{2} } \right\}$$

Before the implementation of MLP, some hyperparameters should be defined by the user, including the number of layers, the number of units per layer, the activation function for each layer, weight decay, learning rate, dropout value, batch size, number of iterations or epochs, and the optimization algorithm. For more information see Waldmann [16] and Pérez-Enciso and Zingaretti [34].

We determined the best set of hyperparameter values by a grid search over a range of values using the whole real dataset. We evaluated the optimization algorithm = [‘SGD’, ‘RMSprop’, ‘Adagrad’, ‘Adadelta’, ‘Adam’, ‘Adamax’, ‘Nadam’], batch size = [32, 64, 128, 256], epochs = [50, 100, 200, 500, 1000], learning rate = [0.001, 0.01, 0.1, 0.2, 0.3], weight decay = [0.00001, 0.0001, 0.001, 0.01], dropout rate = [0.1, 0.2, 0.3, 0.4], units = [8, 16, 32, 64, 128], and layers = [1, 2, 3]. The configuration with the highest prediction accuracy (smaller root mean-squared error) was optimization algorithm = ’SGD’, batch size = 32, epochs = 200, learning rate = 0.01, weight decay = 0.00001, dropout rate = [0.1, 0.1], units = [64, 32] and hidden layers = 2. The nonlinear activation function for the first hidden layer was the rectifier linear unit (“ReLU”) and for the second hidden layer it was “softReLU”. The momentum hyperparameter was considered as 0.5. As a rule of thumb, the more data are available, the smaller dropout value is required. In general, the total number of weights in the hidden layers should be at most 1/2 of the training sample size. MLP was fitted with MXNet package [35] in the R language/environment, version 3.6.1 [24].

### Convolutional neural network

Basically, a CNN [36, 37] is a specialized kind of neural network, where some spatially invariant patterns among the inputs are expected, for example linkage disequilibrium between nearby SNPs in the case of genomic data. As opposed to MLP, where hidden layers are only composed of fully connected layers, in CNN the hidden layers consist of convolutional layers, pooling layers, and fully connected layers. During the training process, a CNN algorithm is able to capture hidden information in the inputs through application of “filters” or kernels in convolution layers. A filter is known as a collection of input values where the weights are the same for all input windows (e.g., SNP windows). A filter is moved across the input matrix, and at each SNP window of the genotype matrix, the CNN computes the local weighted sum and returns an output value. The learned filter moves to the right side of the genotype data with a certain window size until it explains the complete width. Then, the filter moves to the beginning of the next row with the same window size and repeats the process until the entire genotype matrix is traversed. To make the filters slightly invariant to small changes in the input and, also, for dimensionality reduction, a pooling layer is added after each convolutional layer. The pooling layer is usually applied to smooth out the results; it consists of merging the filter outputs of the previous convolutional layer by taking the mean, maximum, or minimum of all values of those filters. Figure 1b represents a general diagram of CNN in a genomic prediction context. For more details on the application of DL in the genomic context, see Bellot et al. [13] and Pérez-Enciso and Zingaretti [34].

The initial values of hyperparameters in our CNN were set based on the papers by Bellot et al. [13] and Ma et al. [14]. Given that those studies used human and plant datasets, we applied the heuristic search of hyperparameters to find the most appropriate values in the back propagation algorithm [38]. The CNN was built with one input layer, one convolutional layer (16 filters), one pooling layer, two fully connected layers (32 and one units, respectively), two dropout layers and one output layer (one unit). Other hyperparameter values used were 200 for number of epochs, 64 for batch size, 0.01 for learning rate, 0.5 for momentum, and 0.00001 for weight decay.

The genotypic matrix was fed to the CNN as input layer. The first convolutional layer extracted the features from the input matrix using 16 filters each with 1 × 5 window size with a stride size of 1 × 3, followed by a max-pooling layer with window size of 1 × 2 and a stride size of 1 × 2. A dropout layer with a rate of 0.3 was assigned to the max-pooling layer. The first fully connected layer with 32 units was used after the convolutional layer with a dropout rate of 0.3. The ReLU activation function was applied in the convolutional layer and a softrelu function was used in the first fully connected layers. The output of the first fully connected layer was then fed to the second fully connected layer with one unit by a softrelu activation function. The output of the second fully connected layer is eventually connected to the output layer using a linear activation function, which presents the individual predicted phenotypic value. The CNN method was fitted with DeepGS package [14] in the R language/environment, version 3.6.1 [24].

### Evaluation of methods

The predictive ability of the different methods in the real dataset was assessed as the correlation between predicted and observed phenotypes $r_{{y,\hat{y}}}$ and the mean squared error of prediction (MSE) using 10 replicates of a five-fold cross validation. In the simulated dataset, predictive ability was evaluated as the correlation between true genotypic values and predicted genomic values, using five replications of a five-fold cross-validation design with 10 iterations. Training and testing sets were the same in both the real data and the simulation datasets.

We compared learning machines using two different types of predictor variables: (i) genotypes at causal loci, and (ii) genotypes at SNPs. In the former case, statistical methods were fitted using the genotypes at causal variants as predictors. In the latter case, to mimic the real SNP data, QTN were excluded from the genotypic matrix and genomic prediction was performed using only the genotypes at SNPs.

It has been argued that machine-learning methods are data hungry; hence we used a larger dataset consisting of 80,000 animals to compare the six methods. Due to the computational burden, only the most complicated simulation scenario consisting of a complex trait with non-additive gene action and 1000 QTN with a clustered distribution was tested.

All analyses were successfully completed on the UF Research Computing HiPerGator supercomputer (https://www.rc.ufl.edu).

## Results

### Real data

**Fig. 2:**
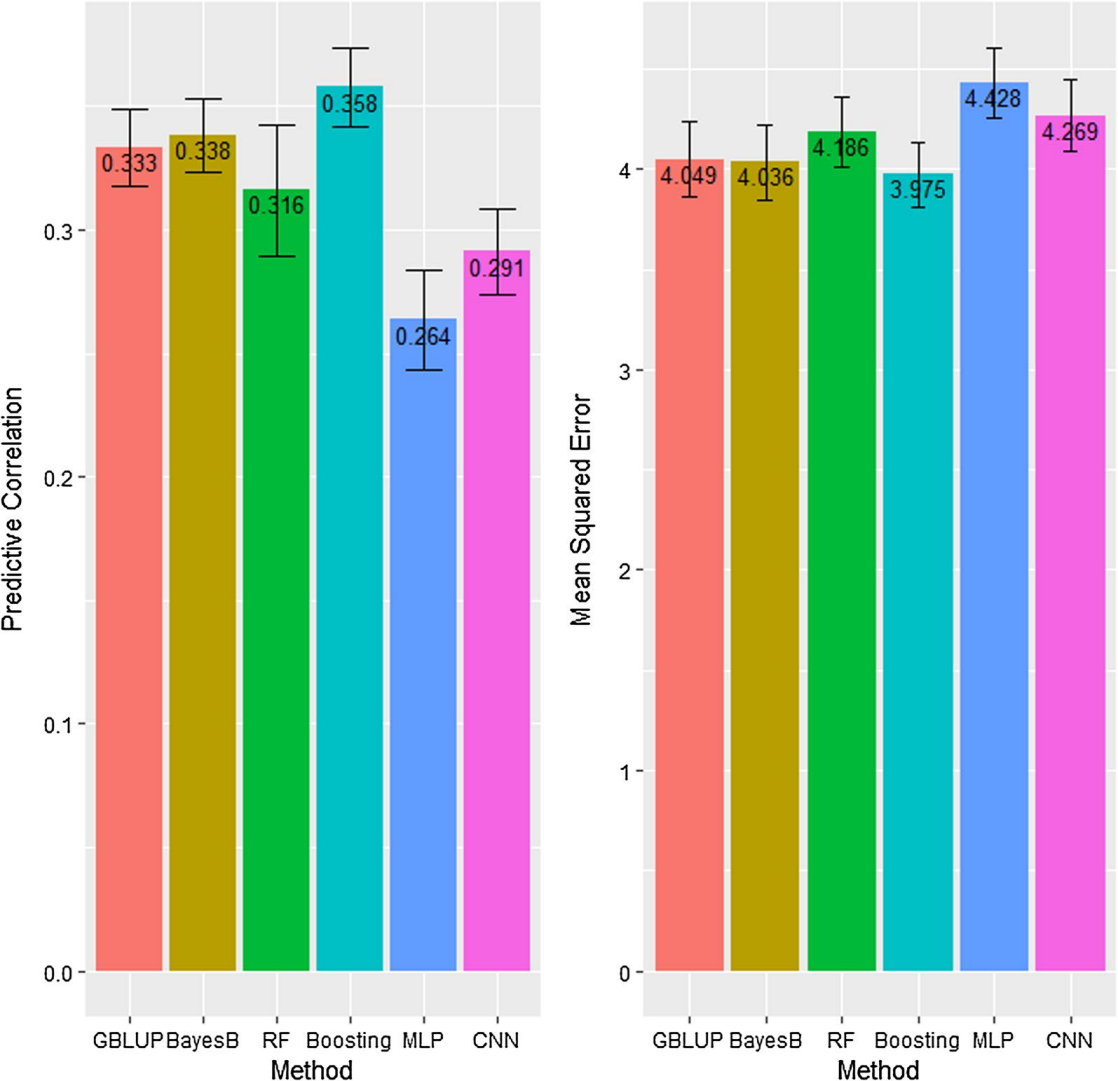
Predictive correlation (left panel) and mean squared error of prediction (right panel) of two conventional statistical methods (GBLUP and Bayes B) and four machine-learning methods including random forests (RF), gradient boosting (Boosting), multilayer perceptron (MLP) and convolutional neural network (CNN) *using a real dataset of sire conception rate records from US Holstein bulls*. The whiskers represent 95% confidence intervals

Figure 2 displays the predictive correlation (left panel) and the mean squared error of prediction (MSE, right panel) of the six prediction methods for the bull (real) dataset. The largest predictive correlation was delivered by GB (0.36) and Bayes B (0.34), followed by GBLUP (0.33), RF (0.32), CNN (0.29) and MLP (0.26). Among the machine-learning approaches, the predictive correlation of CNN was 12% greater than for MLP, but 10% lower than for RF. Although predictive correlation is a simple way of measuring predictive ability, MSE is a preferred metric because it considers both prediction bias and variance. In this sense, Boosting and Bayes B delivered the lowest MSE, followed by GBLUP, RF, CNN and MLP. Figure S1 [see Additional file 1: Figure S1] shows the trend of MSE in the training and validation sets over iterations for MLP; this graph clearly shows that overfitting was not an issue.

### Simulation dataset

We investigated the effect of gene action, number of QTN and QTN distribution across the genome, and of sample size, on the predictive ability of the different methods considered. We used two sets of predictors: (i) genotypes at causal loci and (ii) genotypes at marker loci.

#### Genotypes at causal loci

**Fig. 3:**
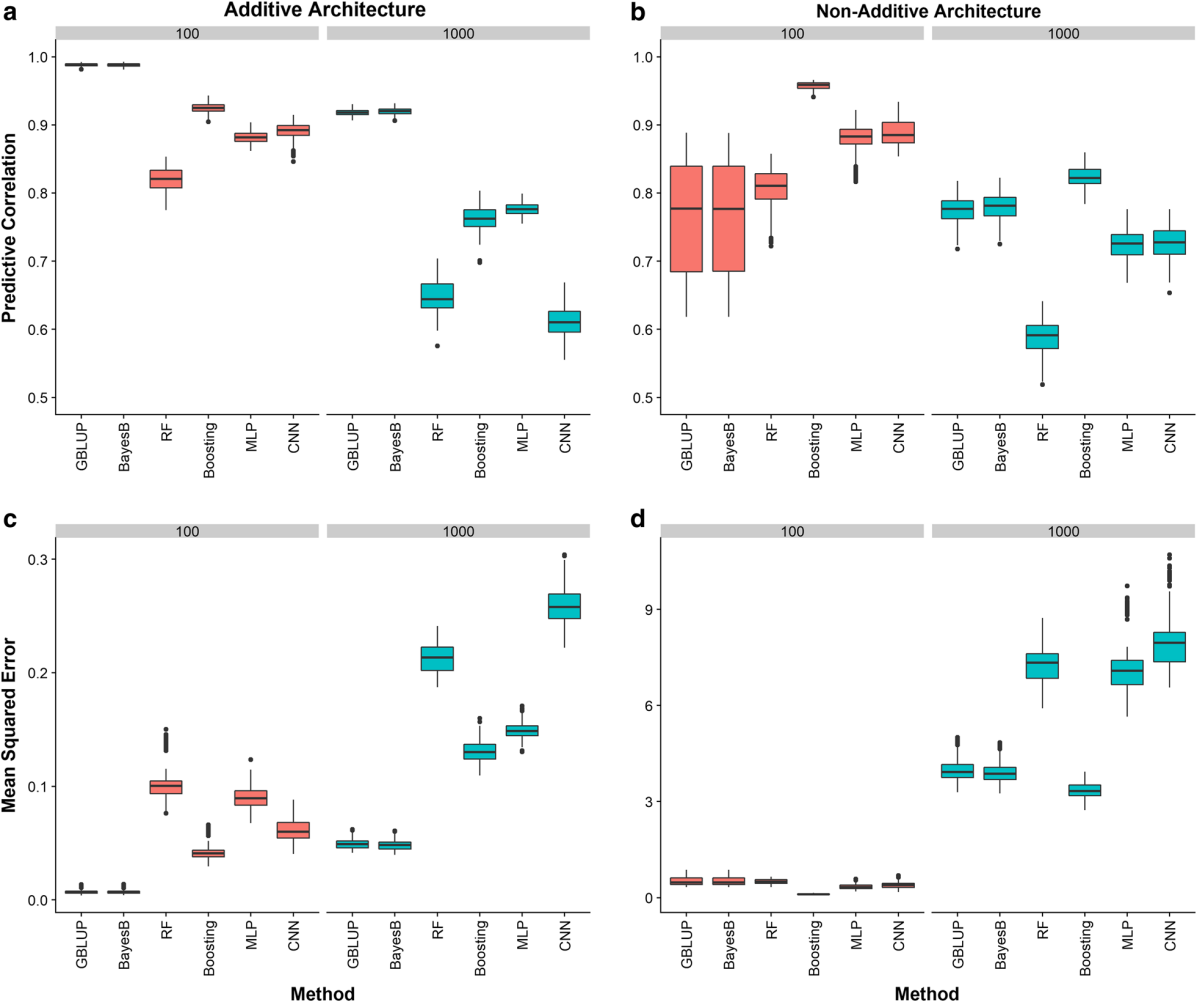
Predictive ability of two conventional statistical methods (GBLUP and Bayes B) and four machine-learning methods including random forests (RF), gradient boosting (Boosting), multilayer perceptron (MLP) and convolutional neural network (CNN) *using genotypes at causal loci*. Predictive ability was evaluated using predictive correlation **a**, **b** and mean squared error **c**, **d**. Different numbers of causal QTN (100 or 1000) and two scenarios of gene action, namely additive and a combination of additive, dominance and epistasis were investigated. The QTN were distributed as clustered across the entire genome.

The predictive ability of different methods using only genotypes at causal loci is shown in Fig. 3. This section illustrates how prediction machines work in an idealized situation where all true QTN are known. When gene action was purely additive, classical statistical methods outperformed machine-learning methods regardless of the number of QTN controlling the trait. Among the machine-learning algorithms, GB (QTN = 100) and GB and MLP (QTN = 1000) attained the best predictive performance (Fig. 3a, c). Interestingly, CNN performed quite well when QTN = 100 but it was the worst method when QTN = 1000. When gene action was non-additive (Fig. 3b, d), GB exhibited the highest predictive performance among the six methods evaluated, regardless of the number of QTN controlling the trait. The predictive performance of the other five methods depended on the number of causal loci: when QTN = 100, the two deep learning algorithms delivered higher predictive correlations and lower MSE values than either GBLUP or Bayes B; however, when the number of QTN was large (QTN = 1000), the two classical statistical methods outperformed both MLP and CNN, and also RF (Fig. 3b). Notably, when QTN were distributed as clustered, the predictive ability of all methods was greater than when the causal loci were distributed randomly across the genome [see Additional file 2: Figures S2, S3, and S4].

Overall, under the same gene action, when the number of causal loci affecting the trait increased, the predictive correlation decreased and MSE increased (Fig. 3 a, b). Clearly, RF did not perform well when there was a large number of causal loci involved, regardless of the gene action.

#### Genotypes at marker loci

**Fig. 4:**
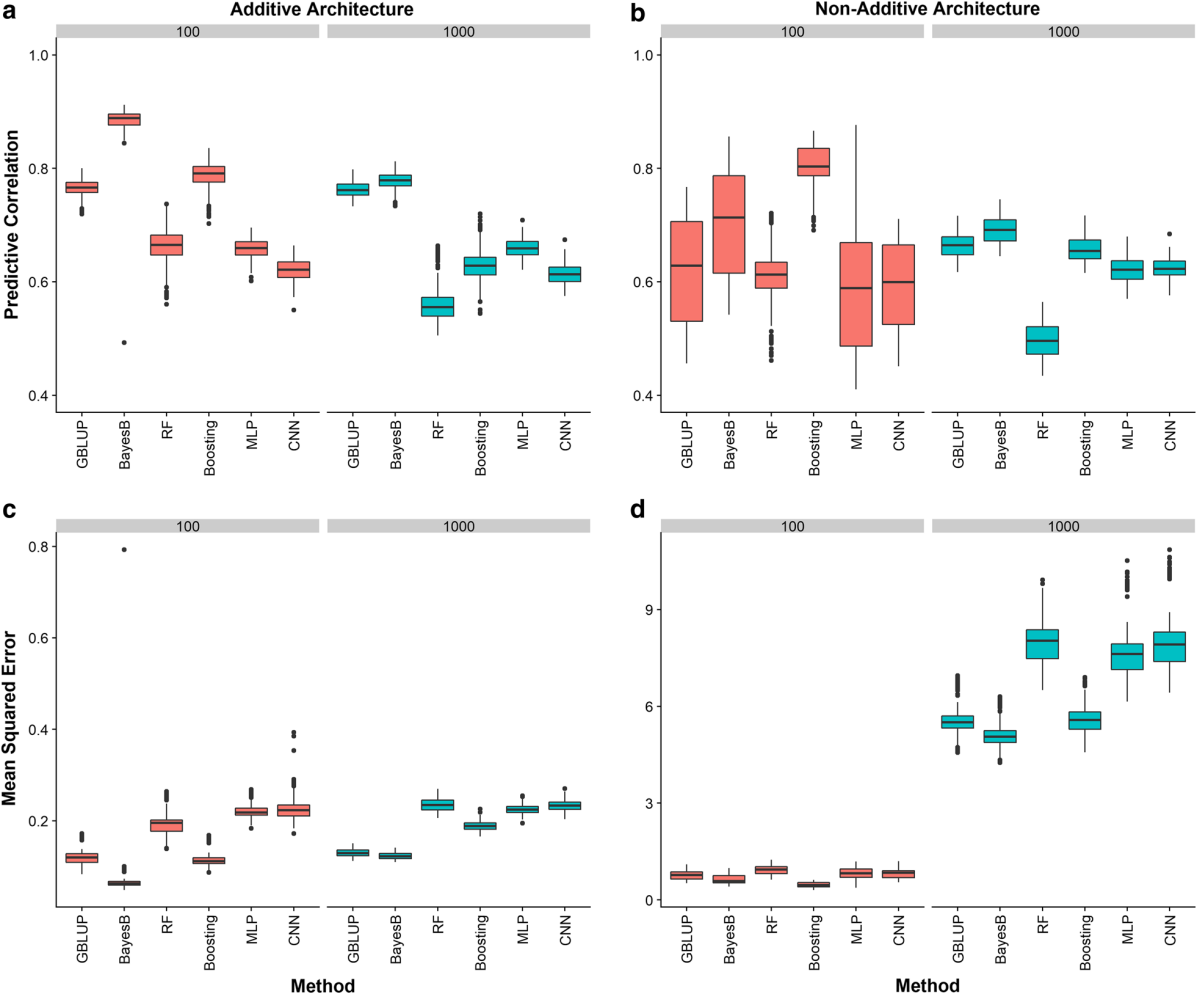
Predictive ability of two conventional statistical methods (GBLUP and Bayes B) and four machine-learning methods including random forests (RF), gradient boosting (Boosting), multilayer perceptron (MLP) and convolutional neural network (CNN) *using genotypes at marker loci*. Predictive ability was evaluated using predictive correlation **a**, **b** and mean squared error **c**, **d**. Different numbers of QTN (100 or 1000) and two scenarios of gene action, namely additive and a combination of additive, dominance and epistasis were investigated. The QTN were distributed as clustered across the genome

The predictive ability of the six different learning machines using genotypes at marker loci under different genetic architectures is shown in Fig. 4. Regardless of the number and distribution of QTN, when gene action was purely additive, Bayes B outperformed both GBLUP and the four machine-learning methods (Fig. 4a, c). Under an additive architecture, GB and MLP were the best machine-learning methods when QTN = 100 and QTN = 1000, respectively. Interestingly, when there were additive, dominance and epistasis effects, the performance of the six methods depended on the number of QTN controlling the trait. When a small number of QTN was simulated, the largest predictive correlation was delivered by GB followed by Bayes B and GBLUP (Fig. 4b). However, when the number of QTN was large, parametric methods outperformed machine-learning methods (Fig. 4b, d).

Notably, machine-learning algorithms were less sensitive to changes in gene action than classical parametric methods. For instance, by moving from additive to non-additive genetic architectures, the predictive ability of Bayes B decreased by about 15%, but the predictive ability of CNN decreased by only 3%. Interestingly, GB exhibited a slightly better predictive performance in the non-additive compared to the additive genetic architecture when the number of QTN was large.

### Sample size

**Fig. 5:**
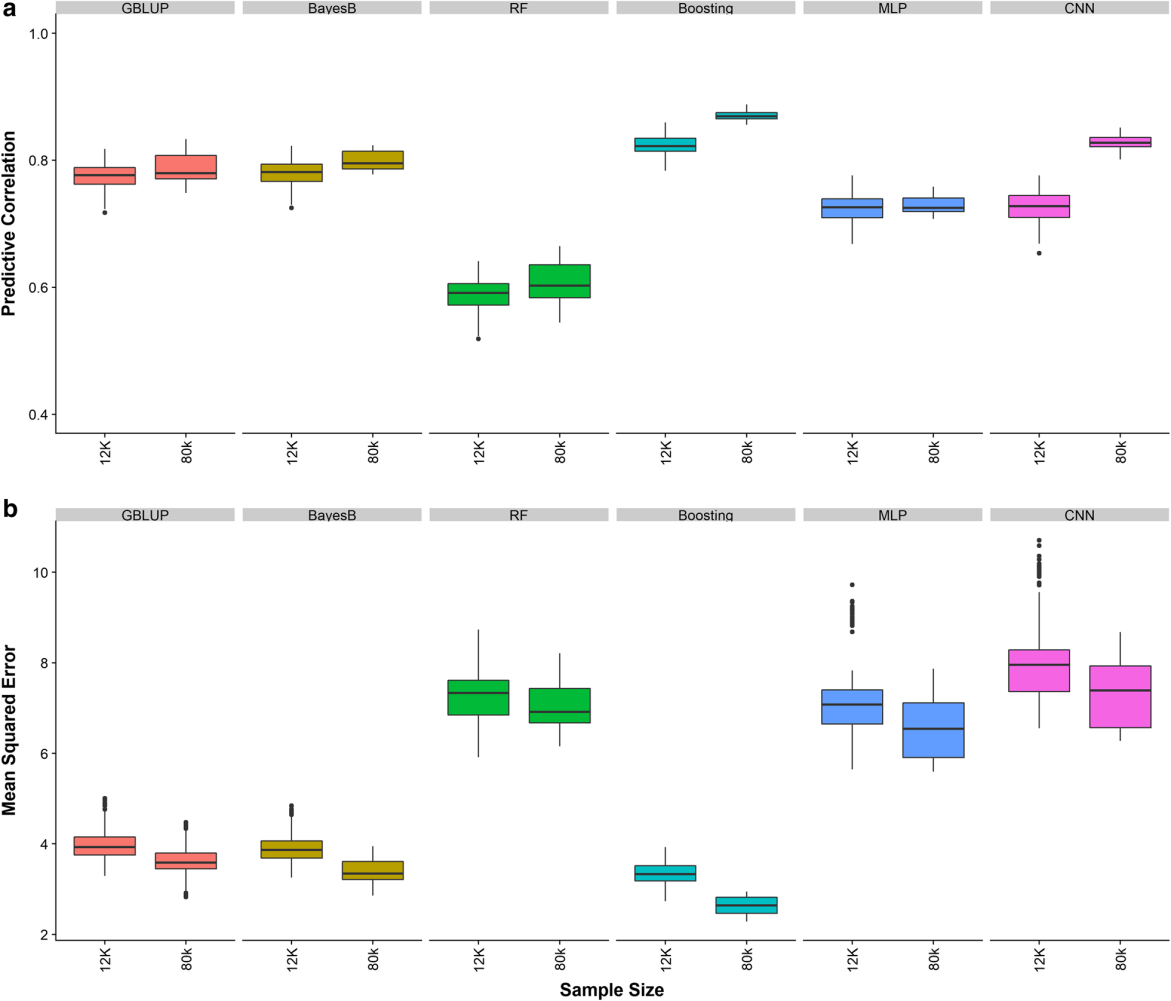
Predictive ability under two sample sizes, 12k and 80k individuals, for two conventional statistical methods (GBLUP and Bayes B) and four machine-learning methods including random forests (RF), gradient boosting (Boosting), multilayer perceptron (MLP) and convolutional neural network (CNN) *using genotypes at causal loci.* Predictive ability was evaluated using predictive correlation **a** and mean squared error **b**. The 1000 causal QTN were distributed as clustered across the genome and gene action was a combination of additive, dominance and epistasis effects

Predictive ability using 80k individuals and 58k SNPs under different genetic architectures is shown in Fig. 5. Due to the computational burden, we explored only the most complex gene action (additive + dominance + epistasis) and 1000 QTN distributed as clustered across the genome. In all cases, the predictive performance increased relative to the performance attained with only 12k individuals. Interestingly, when 12k individuals were used, the parametric methods were better than the deep learning methods. However, when the sample size was large (n = 80k), CNN outperformed classical statistical methods in terms of predictive correlation (0.81 vs. 0.79) but not in MSE. The gain in predictive correlation via increasing sample size was more pronounced for deep learning than for parametric methods, e.g., 12% for CNN but only 3% for Bayes B. Similarly, the decrease in MSE by moving from 12k to 80k individuals was 0.68 for CNN and 0.50 for Bayes B.

## Discussion

Our main objective in this study was to evaluate the performance of deep learning algorithms for prediction of complex phenotypes. Sire conception rate in cattle is a complex trait and previous studies have reported both additive and non-additive effects on this trait [39, 40]. Since the genetic architecture underlying SCR is unclear, we also investigated the performance of learning algorithms using simulated traits under simple (purely additive) and more complex conditions (joint effects of additive, dominance and epistatic interactions). These two architectures served as a ‘stress test’, since parametric methods may not always work well with complex genetic architectures.

Here, we used a simple additive model in GBLUP and Bayes B for the analysis of traits with non-additive effects. It has been reported that a statistical model combining additive and epistatic effects performs better than a simple additive model for the analysis of quantitative traits with epistatic architecture [41]. Machine-learning methods can capture non-additive effects without any assumptions about gene action. Furthermore, differences in predictive ability among machine-learning algorithms could be observed because of the intrinsic ways in which marker information is processed by various methods [42].

Our results confirmed that the performance of prediction machines depends on the genetic architecture of the trait. Under pure additive actions, conventional statistical methods outperformed machine-learning approaches. However, when there was non-additive action, predictive ability depended on the number of loci controlling the trait. When the trait was controlled by a small number of loci with complex gene actions, machine-learning algorithms performed similarly or even better than conventional statistical models. Simulation results showed that GB had some advantages over other methods under complex gene action and with a small number of QTN (*n* = 100) involved. It has been argued that, for complex traits controlled by many genes with epistatic interaction effects, machine-learning methods are promising and have potential to outperform parametric statistical methods [11, 42–44]. In contrast, we found that machine-learning methods might be suitable for the prediction of traits with a small number of QTN with strong epistatic effects provided that loci are clustered, as observed in Waldmann [16].

When prediction of additive genetic values is the primary interest, there may not be any benefit from using methods that capture interactions, as they do not contribute much, if at all, to genetic variance. Nevertheless, when phenotypic predictions are desired, such as predicting semen fertility, machine-learning algorithms incorporating interaction effects may perform better than models capturing only additive effects [45]. It has also been demonstrated that deep learning algorithms may be useful for predicting individual genotypic value for traits that are affected by genotype-by-environment interactions [15].

In our simulations, when the number of QTN affecting the trait increased from 100 to 1000, the predictive performance of all methods declined. An explanation may be that a larger sample size is needed to capture the tiny effects of a large number of additive, dominance and interaction effects. We had hypothesized that application of DL for predicting complex traits controlled by a large number of loci would require a large sample size. Indeed, larger sample sizes improved the predictive ability of machine-learning methods, especially GB and CNN, under non-additive genetic architectures. However, a larger sample size did not translate into a marked improvement in prediction accuracy of the parametric methods. Given that the cost of genotyping and sequencing has decreased remarkably over the last decade, which allows now to perform studies with larger sample sizes, the identification of the most accurate and applicable prediction machine is important.

We simulated scenarios in which QTN were either randomly distributed across the genome or clustered in particular genomic regions. There is growing evidence that supports the idea that QTN may be located in clusters. For example, Wood et al. [46] found 697 significant hits for human height distributed in 423 distinct clusters in the human genome. Clustering of QTN in specific genomic regions could be due to selection for particular combinations of favorable alleles or because of sharing common regulatory elements [47]. Notably, we found that the performance of the different predictive machines was better when QTN were clustered. Similarly, Bellot et al. [13] found that significant SNPs in clusters delivered better predictive performance than significant SNPs uniformly distributed over the genome.

Whole-genome prediction differs in a very important way from image or speech recognition tasks [33]. Complex traits are multifactorial, where environmental factors may differ from individual to individual, and epigenetic marks can affect performance, so that the genotype of an individual may not provide sufficient information to predict phenotypes accurately [48]. However, there are some similarities between genomics and other domains, for instance genotype–phenotype associations can be viewed as a landscape. This landscape may have extremely steep valleys, where small perturbations in genotype give rise to vastly different phenotypes [49]. It may also have large plateaus, where seemingly unrelated genotypes yield an equivalent phenotype.

There are some caveats with the application of machine learning in genomics: (1) machine-learning and statistical methods both can be used in a prediction context, but machine-learning methods, and DL methods in particular, are not useful for inference [50]; (2) researchers are often more interested in the biological meaning of a predictive model than in its predictive accuracy, and the ‘black box’ nature of machine-learning methods, especially neural networks with a large number of layers and units, can inhibit interpretation; (3) the loss function when studying association of genotypes with phenotypes may present local minima and maxima, so finding a global optimum is probably difficult; (4) as the number of input variables increases, the number of weights to be learned in a neural network increases exponentially, so the chance of overfitting also increases; (5) the design of a proper network requires considerable knowledge; for instance, in CNN finding the appropriate hyper-parameters for each of the convolutional, pooling, and fully connected layers is very challenging, especially in terms of understanding the biological significance [14].

## Conclusions

We trained two conventional statistical models, GBLUP and Bayes B, along with two tree ensemble learning methods, GB and RF, in order to compare model predictive ability against two common deep learning algorithms, MLP and CNN. For a complex phenotype such as sire conception rate, the best predictive performance was obtained using GB. We also investigated the performance of deep learning methods in a wide range of genetic architectures of simulated complex traits with two different sample sizes. When the genetic architecture of a trait was purely additive, classical parametric methods outperformed machine-learning methods. However, when the gene action was non-additive, GB exhibited the best predictive performance. DL algorithms worked well in the non-additive setting provided that a large sample size was available, but their performance was not entirely consistent. Overall, GB is a robust method in genomic prediction of complex traits and DL does not appear to be a panacea for genome-enabled prediction of complex traits.

## Supplementary information

**Additional file 1: Figure S1.** Visualization of the performance of multilayer perceptron (MLP) algorithm based on the mean squared error during the training process.

**Additional file 2: Figure S2.** Predictive ability of two conventional statistical methods (GBLUP and Bayes B) and four machine-learning methods including random forests (RF), gradient boosting (Boosting), multilayer perceptron (MLP) and convolutional neural network (CNN) *using genotypes at causal loci*. Predictive ability was evaluated using predictive correlation (**a**, **b**) and mean squared error (**c**, **d**). Different numbers of QTN (100 or 1000) and two scenarios of gene action, namely purely additive (left panel) and a combination of additive, dominance and epistasis (right panel) were investigated. The QTN were randomly distributed across the genome. **Figure S3.** Predictive ability of two conventional statistical methods (GBLUP and Bayes B) and four machine-learning methods including random forests (RF), gradient boosting (Boosting), multilayer perceptron (MLP) and convolutional neural network (CNN) *using genotypes at marker loci*. Predictive ability was evaluated using predictive correlation (**a**, **b**) and mean squared error (**c**, **d**). Different numbers of QTN (100 or 1000) and two scenarios of gene action, namely purely additive (left panel) and a combination of additive, dominance and epistasis (right panel) were investigated. The QTN were randomly distributed across the genome. **Figure S4.** Predictive ability under two sample sizes, 12 k and 80 k individuals, for two conventional statistical methods (GBLUP and Bayes B) and four machine-learning methods including random forests (RF), gradient boosting (Boosting), multilayer perceptron (MLP) and convolutional neural network (CNN) *using genotypes at marker loci*. Predictive ability was evaluated using predictive correlation (**a**, **b**) and mean squared error (**c**, **d**). The 1000 causal QTN were distributed as clustered across the genome and gene action was a combination of additive, dominance and epistasis effects

## Data Availability

The phenotypic data are available at the website of the Council on Dairy Cattle Breeding (https://www.uscdcb.com/). The genotypic data are available upon reasonable request to the Cooperative Dairy DNA Repository. The simulation script is available from the corresponding author upon reasonable request.
