# Supplementary figures and images for: Deep learning versus parametric and ensemble methods for genomic prediction of complex phenotypes
Source: Genet Sel Evol. 2020 Feb 24;52:12. doi: 10.1186/s12711-020-00531-z (PMC7038529; doi:10.1186/s12711-020-00531-z)

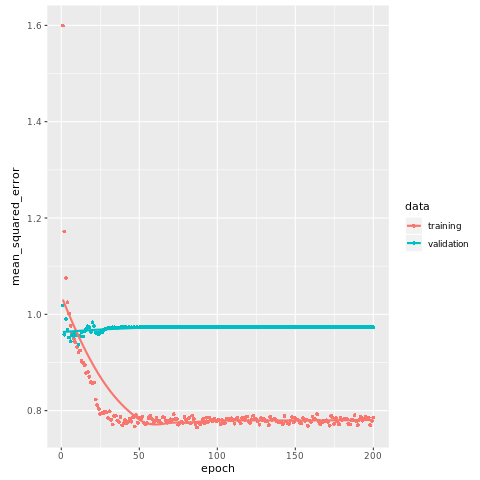

Supplement: Supplementary file 1 — Additional file 1: Figure S1. Visualization of the performance of multilayer perceptron (MLP) algorithm based on the mean squared error during the training process. [file 12711_2020_531_MOESM1_ESM.tiff]

Figure S2

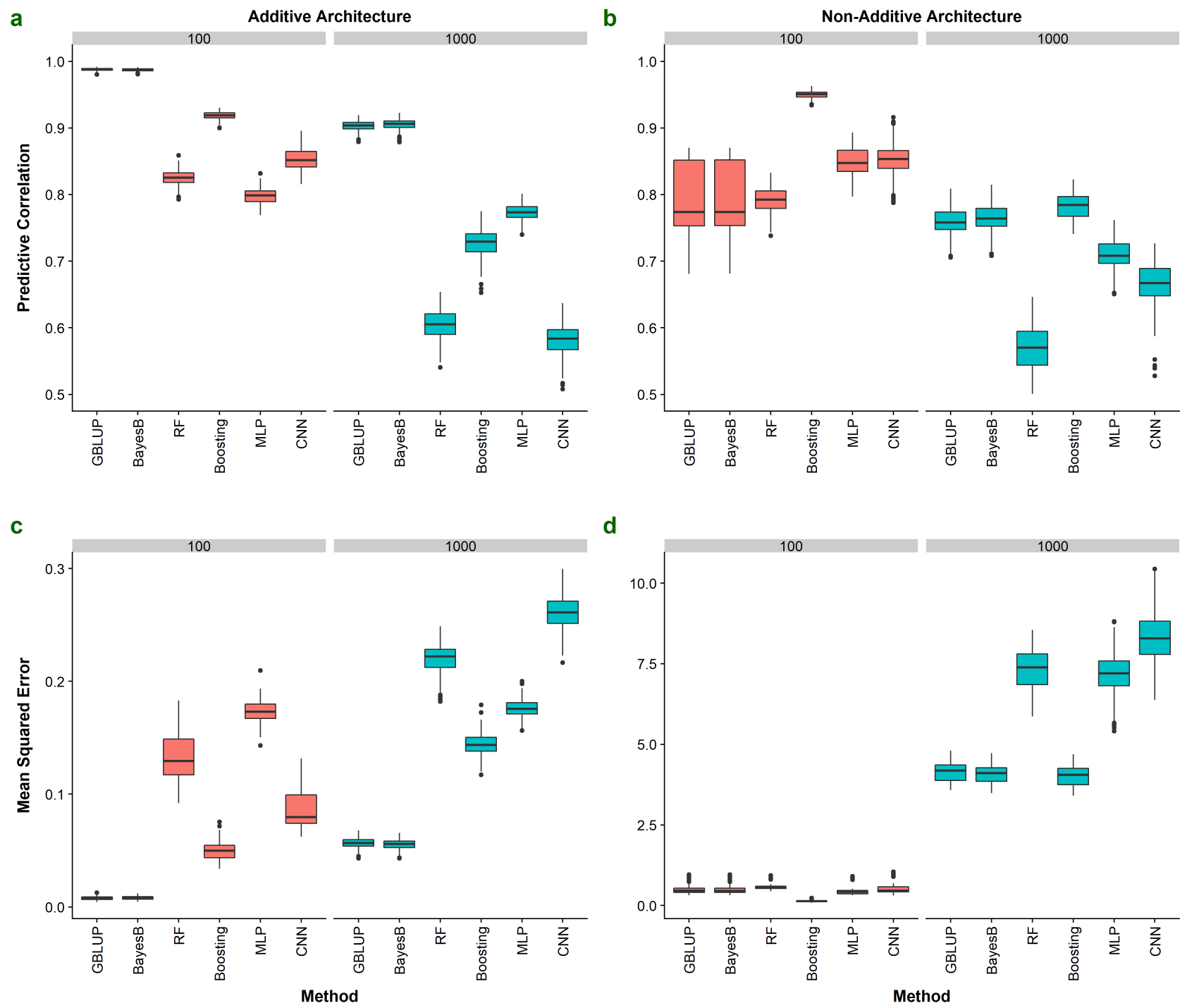

Figure S3

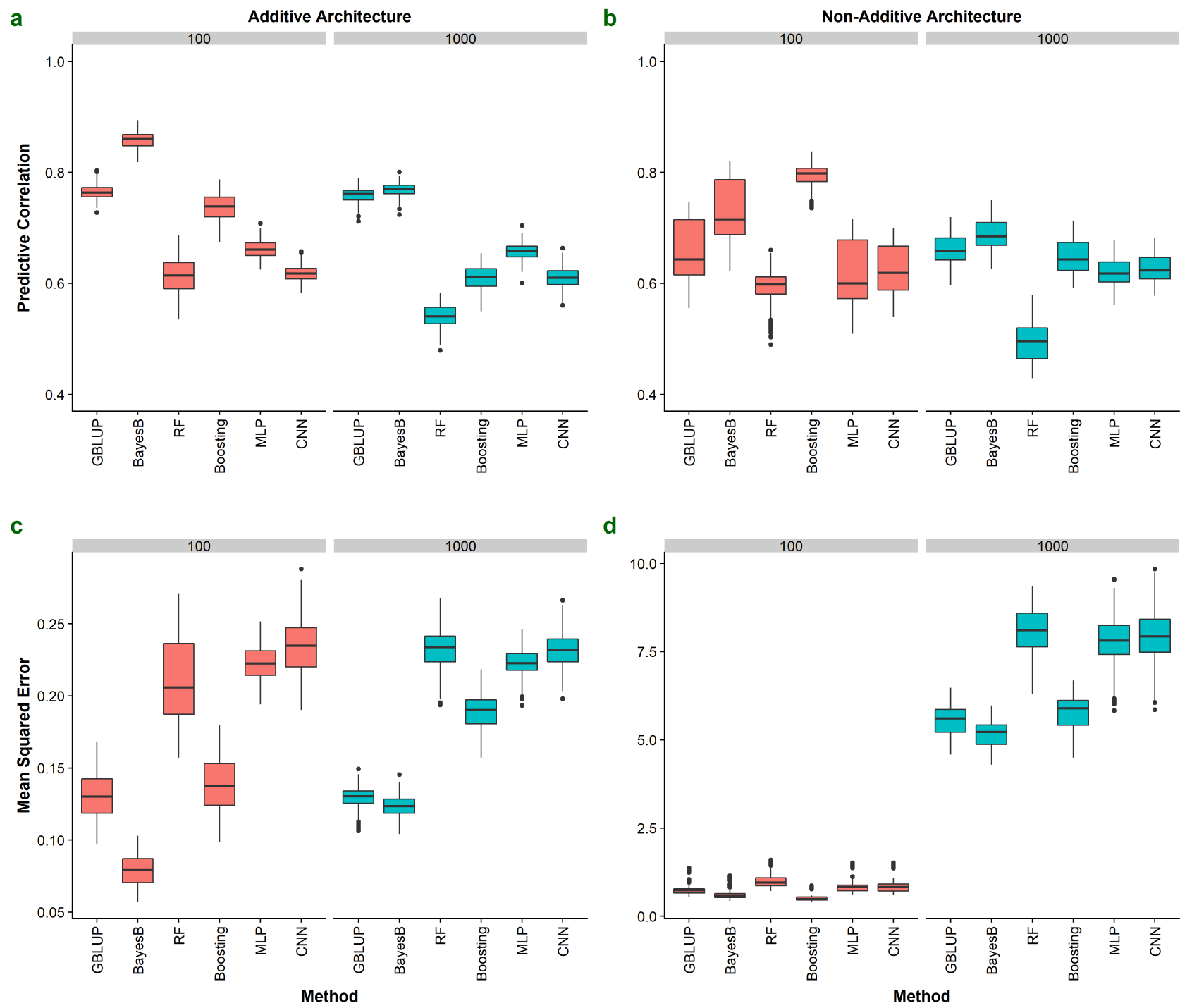

Figure S4

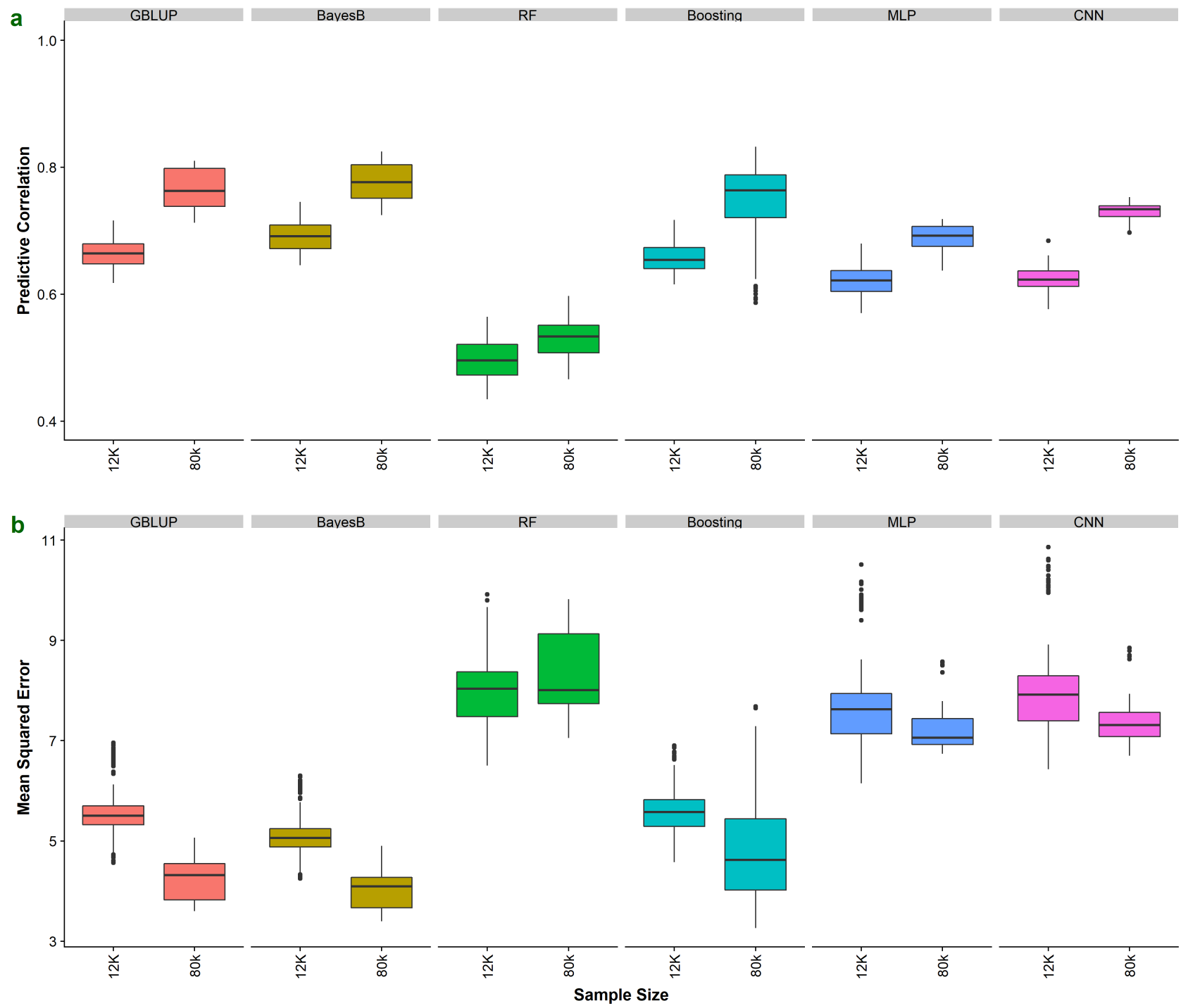

Supplement: Supplementary file 2 — Additional file 2: Figure S2. Predictive ability of two conventional statistical methods (GBLUP and Bayes B) and four machine-learning methods including random forests (RF), gradient boosting (Boosting), multilayer perceptron (MLP) and convolutional neural network (CNN) using genotypes at causal loci. Predictive ability was evaluated using predictive correlation (a, b) and mean squared error (c, d). Different numbers of QTN (100 or 1000) and two scenarios of gene action, namely purely additive (left panel) and a combination of additive, dominance and epistasis (right panel) were investigated. The QTN were randomly distributed across the genome. Figure S3. Predictive ability of two conventional statistical methods (GBLUP and Bayes B) and four machine-learning methods including random forests (RF), gradient boosting (Boosting), multilayer perceptron (MLP) and convolutional neural network (CNN) using genotypes at marker loci. Predictive ability was evaluated using predictive correlation (a, b) and mean squared error (c, d). Different numbers of QTN (100 or 1000) and two scenarios of gene action, namely purely additive (left panel) and a combination of additive, dominance and epistasis (right panel) were investigated. The QTN were randomly distributed across the genome. Figure S4. Predictive ability under two sample sizes, 12 k and 80 k individuals, for two conventional statistical methods (GBLUP and Bayes B) and four machine-learning methods including random forests (RF), gradient boosting (Boosting), multilayer perceptron (MLP) and convolutional neural network (CNN) using genotypes at marker loci. Predictive ability was evaluated using predictive correlation (a, b) and mean squared error (c, d). The 1000 causal QTN were distributed as clustered across the genome and gene action was a combination of additive, dominance and epistasis effects [file 12711_2020_531_MOESM2_ESM.pdf]
